# IMMUNEPOTENT CRP induces cell cycle arrest and caspase-independent regulated cell death in HeLa cells through reactive oxygen species production

**DOI:** 10.1186/s12885-017-3954-5

**Published:** 2018-01-03

**Authors:** Ana Carolina Martínez-Torres, Alejandra Reyes-Ruiz, Milena Benítez-Londoño, Moises Armides Franco-Molina, Cristina Rodríguez-Padilla

**Affiliations:** Universidad Autónoma de Nuevo León, Facultad de Ciencias Biológicas, Laboratorio de Inmunología y Virología, 66455 Monterrey, Mexico

**Keywords:** I-CRP, Bovine dyalisable leukocyte extract, bDLE, Cell death, Cervical cancer, Immunotherapy, Transfer factor, Immunomodulator, Apoptosis

## Abstract

**Background:**

Regulated cell death (RCD) is a mechanism by which the cell activates its own machinery to self-destruct. RCD is important for the maintenance of tissue homeostasis and its deregulation is involved in diseases such as cervical cancer. IMMUNEPOTENT CRP (I-CRP) is a dialyzable bovine leukocyte extract that contains transfer factors and acts as an immunomodulator, and can be cytotoxic to cancer cell lines and reduce tumor burden in vivo*.* Although I-CRP has shown to improve or modulate immune response in inflammation, infectious diseases and cancer, its widespread use has been limited by the absence of conclusive data on the molecular mechanism of its action.

**Methods:**

In this study we analyzed the mechanism by which I-CRP induces cytotoxicity in HeLa cells. We assessed cell viability, cell death, cell cycle, nuclear morphology and DNA integrity, caspase dependence and activity, mitochondrial membrane potential, and reactive oxygen species production.

**Results:**

I-CRP diminishes cell viability in HeLa cells through a RCD pathway and induces cell cycle arrest in the G2/M phase. We show that the I-CRP induces caspase activation but cell death induction is independent of caspases, as observed by the use of a pan-caspase inhibitor, which blocked caspase activity but not cell death. Moreover, we show that I-CRP induces DNA alterations, loss of mitochondrial membrane potential, and production of reactive-oxygen species. Finally, pretreatment with N-acetyl-L-cysteine (NAC), a ROS scavenger, prevented both ROS generation and cell death induced by I-CRP.

**Conclusions:**

Our data indicate that I-CRP treatment induced cell cycle arrest in G2/M phase, mitochondrial damage, and ROS-mediated caspase-independent cell death in HeLa cells. This work opens the way to the elucidation of a more detailed cell death pathway that could potentially work in conjunction with caspase-dependent cell death induced by classical chemotherapies.

**Electronic supplementary material:**

The online version of this article (10.1186/s12885-017-3954-5) contains supplementary material, which is available to authorized users.

## Background

Regulated cell death (RCD) is a physiological mechanism by which the cell activates its own machinery to self-destruct. It is important for the maintenance of tissue homeostasis and its deregulation induces diseases such as cancer. Among the different types of cancer, cervical cancer remains one of the leading causes of women death worldwide [1]. Although several approaches such as immune therapy with cytokines, polyamine synthesis inhibitors, individual micronutrient supplementation and pharmaceutical agents exist, they have shown a limited success [2, 3]. And while chemotherapy and radiotherapy are front-line treatments against this and other types of cancer, they cause important side effects. These are reasons why the development of new therapies to improve existing treatments is a major challenge. Natural-derived products have recently attained a lot of interest for their ability to modulate the signaling pathways involved in cancer proliferation or for their protective potential in radiotherapy and chemotherapy [4]. Some such natural-derived products are dialyzable leukocyte extracts (DLE), which are mixtures of low molecular weight substances (<12 kDa) released from disintegrated leukocytes of human, bovine, or other species’ blood or tissue [5, 6]. DLEs have been used as therapeutic agents in the treatment of a broad spectrum of diseases, usually related with the immune system [7], including cancer, where they have shown to improve the quality of patients’ lives [8, 9].

Results obtained in our laboratory show that a type of bovine DLE obtained from disintegrated spleen, IMMUNEPOTENT CRP**©** (I-CRP), is cytotoxic to several cancer cell lines [6]. In the MCF-7 cell line, I-CRP inhibited cell growth, suppressed the DNA-binding activity of AP-1, decreased c-Jun protein expression [8] and modulated the mRNA expression of NFATx, NFATc, NFκB, c-Jun, c-Fos, p53, bag-1, c-myc, bax, bcl-2 [6]. It has also prevented murine melanoma cell growth and diminished VEGF release [10]. However, the mechanisms by which I-CRP exerts these effects and the type of cell death activated in these or other cell lines are still unknown. The limited information of its molecular action mechanisms has limited its widespread use.

The purpose of this study was to analyze the molecular pathways by which I-CRP exerts its cytotoxicity. We used the cervical cancer-derived HeLa cell line to further characterize its mechanism of cytotoxicity. We found that I-CRP induces caspase-independent but ROS-dependent cell death, loss of mitochondrial membrane potential, DNA fragmentation and condensation, and cell cycle arrest in HeLa cells.

## Methods

### Cell culture

Human cervix adenocarcinoma HeLa (ATCC® CCL-2™) and human cervix squamous carcinoma SiHa (ATCC® HTB-35™) cells were obtained from the American Type Culture Collection and maintained in a humidified incubator containing 5% CO_2_ at 37 °C. HeLa cells were cultured in DMEM-F12 supplemented with 10% fetal bovine serum (FBS) and 1% penicillin-streptomycin (GIBCO). Cells were routinely grown in plastic tissue-culture dishes (CORNING).

### Cell death induction and inhibition

The bovine dialyzable leukocyte extract, IMMUNEPOTENT CRP**©** (I-CRP), was produced as described previously [6, 11], and was dissolved in media. One unit of I-CRP is defined as the product obtained from 1 × 10^8^ leukocytes [11, 12]. Etoposide and QVD.opH (BioVision) were dissolved in DMSO. N-acetyl-L-cysteine (NAC) and H_2_O_2_ were dissolved in water. For cell death induction, cells were seeded and incubated with the indicated concentration of I-CRP, etoposide, or H_2_O_2_ at the indicated times. For cell death inhibition, QVD.oph or NAC were added 30 min before I-CRP, etoposide, or H_2_O_2_ treatment. All stock solutions were wrapped in foil and stored at −20 °C. All reagents were from SIGMA-ALDRICH, unless otherwise stated.

### Cell viability assessment

Cell growth inhibition was determined by measuring 3-(4,5-dimethylthiazol-2-yl)-2,5-diphenyltetrazolium bromide (MTT) dye absorbance by living cells, as previously described [6]. In brief, 5 × 10^3^ cells per well were seeded in 96-well microtiter plates for MTT assays. After exposure to IMMUNEPOTENT CRP for 4, 8, 16, 24, 48, and 72 h, twenty microliters of MTT solution (2 mg/ml in PBS) were added to each well. The plates were incubated for 3 additional hours at 37 °C, after which the MTT solution in the medium was aspirated and 200 μl of DMSO were added to each well to solubilize the formazan crystals formed in the viable cells. The optical density was measured at 570 nm using a microplate reader (Synergy2, Biotek).

### Cell death analysis

Cell death was determined by staining cells with annexin-V-allycophalloidin (APC, BD) and propidium iodide (PI), as previously described [13]. In brief, 2 × 10^5^ cells were seeded in 24-well plates (Corning) and were incubated with IMMUNEPOTENT CRP for 24 h, with or without incubation with QVD.oph. Cells were then detached and washed twice with PBS and then resuspended in 200 μl of binding buffer (10 mM HEPES/NaOH pH 7.4, 140 mM NaCl, 2.5 mM CaCl2). Cells were then stained and subsequently assessed with a flow cytometer (Becton Dickinson, BDAccury6) and analyzed using FlowJo Software.

### Cell counting and blue trypan staining

Time-lapse cellular cytotoxicity induced by 1.25 U/mL of I-CRP treatment was measured with trypan blue exclusion assay. Briefly, 1 × 10^4^ cells were seeded into 96-well plates and treated with I-CRP for 16, 24, 48, and 72 h, or left without treatment, as a control. After the incubation period, the cells were harvested and washed twice with PBS, and the cell pellet was then resuspended with 0.5 mL PBS. Then, 20 μL of suspension was mixed with equal volume of 0.4% trypan blue and was count using a Neubauer chamber (Superior Marienfeld) and a clear-field microscopy (Primostar Zeiss). Each I-CRP and control was assayed three times in quintuplicate. Total cells were counted, and the percentage of trypan blue positive cells was obtained.

### Cell morphology assessment

HeLa cells were cultured in 24-well plates and left untreated or incubated for 16, 24, 48, and 72 h with I-CRP. After the incubation time, plates were observed in an inverted microscope (NIKON TS100) and pictures were obtained with an Infinity1 (Lumenera) camera.

### Cell cycle analysis

Cell cycle distributions were determined by PI staining. In brief, 5 × 10^5^ cells in 6-well dishes (CORNING) were incubated with I-CRP at different times (4, 8, 16, 24, 48 h) and concentrations (1, 1.25, and 2 U/mL). Cells were then washed with PBS and fixed in 70% ethanol. Cells were washed again with PBS, then incubated with PI (10 μg/ml) with simultaneous RNase treatment at 37 °C for 30 min. Cell DNA contents were measured using a flow cytometer (Becton Dickinson, BDAccury6), and analyzed using FlowJo Software.

### Nuclear assessment

For DNA degradation we analyzed the SubG1 population obtained from cell cycle analysis, using a flow cytometer (Becton Dickinson, BDAccury6), and analyzed using FlowJo Software. For chromatin condensation we did Hoechst staining. In brief 10 × 10^5^ cells were incubated in 6-well plates, then treated with I-CRP and then were washed in PBS and fixed with paraformaldehyde 4%. We stained the cells for 5 min using 5 μg/ml Hoechst 33,342 (SIGMA-ALDRICH). Cells were then washed with PBS, observed using a fluorescence microscope (OLYMPUS IX70) and analyzed with Image-J software.

### Caspase analysis

Caspase activity was measured using Caspase 3 (active) FITC staining kit (ABCAM). In brief, 5 × 10^5^ cells in 6-well dishes (CORNING) were incubated with IMMUNEPOTENT CRP alone or co-cultured with QVD.oph. Cells were then recuperated and stained following the manufacturer’s instructions. Caspase activity was measured using a flow cytometer (Becton Dickinson, BDAccury6) and analyzed using FlowJo Software.

### Mitochondrial membrane potential analysis

Mitochondrial membrane potential was measured using TMRE (125 nM) (SIGMA-ALDRICH). In brief, 5 × 10^5^ cells in 6-well dishes (CORNING) were incubated as indicated. Cells were then recuperated, washed with PBS, stained, incubated at 37 °C for 30 min, and measured using a flow cytometer (Becton Dickinson, BDAccury6) and analyzed using FlowJo Software. For fluorescence microscopy, cells were washed in PBS after treatment, stained, and incubated at 37 °C for 30 min. Cells were then washed with PBS and observed using a fluorescence microscope (OLYMPUS IX70).

### ROS production analysis

ROS generation was measured using DCFDA (2.5 μM) (Invitrogen). In brief, 5 × 10^5^ cells in 6-well dishes (CORNING) were incubated as indicated. Cells were then recuperated, washed with PBS, stained, incubated at 37 °C for 30 min, and measured using a flow cytometer (Becton Dickinson, BDAccury6) and analyzed using FlowJo Software. For fluorescence microscopy cells were washed in PBS after treatment, stained, and incubated at 37 °C for 30 min. Cells were then washed with PBS and observed using a fluorescence microscope (OLYMPUS IX70).

### Statistical analysis

The results given in this study represent the mean of at least four independent experiments done in triplicate (mean ± SD). The data was analyzed using GraphPad Prism (San Diego, CA, USA). Statistical analysis was done using paired student T-test. The statistical significance was defined as *p* < 0.05.

## Results

### IMMUNEPOTENT CRP-treatment diminishes cell viability in HeLa cells

**Fig. 1:**
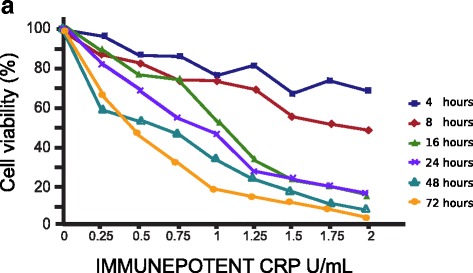
Concentration and time effect of I-CRP exposure on HeLa cell viability. After HeLa cells were treated with various concentrations (0.25, 0.5, 0.75, 1.0, 1.25, 1.5, 1.75, 2 U/mL) of I-CRP for different times (4, 8, 16, 24, 48, 72 h), cell viability was measured by MTT assay. The percentages refer to relative cell viability represented as percentage of control (non-treated cell viability = 100%)

I-CRP has been shown to suppress cell viability in several tumor cell lines [6, 10]. However, its effect on cervical cancer-derived HeLa cell line, has not been assessed, thus, we determined the effect of I-CRP on these cells. IMMUNEPOTENT CRP decreased the viability of HeLa cells in a dose- and time-dependent-manner (Fig. 1). We observed low cytotoxic effects after 4 h and 8 h of treatment, and observed that after 16 h we could detect a considerable diminution of cell viability, which continued to decrease after 24, 48, and 72 h. The cytotoxic concentration that decreased the viability of 50% of the cells (CC50) after 8 h is 2 U/mL, after 16 h and 24 h of treatment is 1 U/mL, after 48 h is 0.75 U/mL, and after a 72 h–treatment it is 0.5 U/mL (Fig. 1).

### IMMUNEPOTENT CRP induces cell death and inhibits cell recovery in HeLa cells

**Fig. 2:**
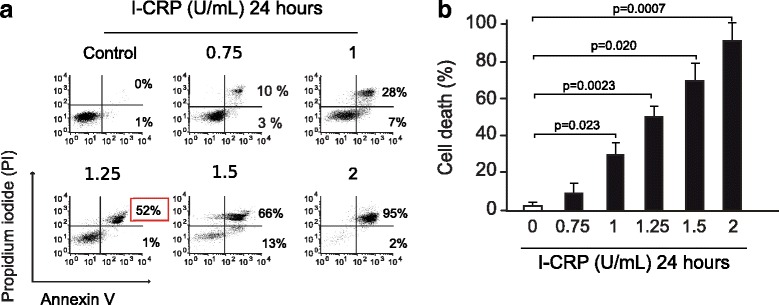
Phosphatidylserine exposure and membrane permeability of HeLa cells after I-CRP exposure. **a** Cell death was measured by flow cytometry through Annexin-V and PI staining in HeLa cells treated with different concentrations (0.75, 1.0, 1.25 1.5, 2 U/mL) of I-CRP for 24 h. The percentages refer to Annexin-V-positive/PI-negative or Annexin-V-positive/PI-positive staining analyzed by flowjo software. **b** Cells were treated and analyzed as in (A) and graphed

**Fig. 3:**
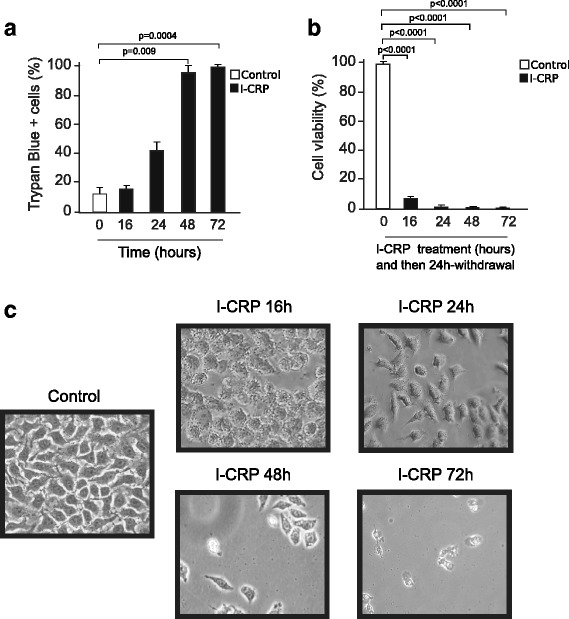
Time-dependent characteristics of cell death induced by I-CRP in HeLa cells. **a** Cell death was measured by trypan blue exclusion after I-CRP treatment (1.25 U/mL) for 16 h, 24 h, 48 h, and 72 h and was analyzed as a percentage of living cells and graphed. **b** Effect on cell viability after withdrawal of I-CRP (1.25 U/mL) administered for 16 h, 24 h, 48 h and 72 h, was measured by MTT assay after 24 h of withdrawal. The percentages refer to relative cell viability represented as % control (non-treated cell viability = 100%). **c** Changes in morphology of HeLa cells induced by treatment with I-CRP (1.25 U/mL) for 16 h, 24 h, 48 h and 72 h, observed in an inverted microscope (NIKON TS100) (20X)

As the MTT assay measures cell metabolism through the capacity of the viable cells to reduce MTT into formazan crystals [14], we further evaluated cell death by assessing phosphatidylserine (PS) exposure (Annexin-V-APC) and membrane permability (propidium iodide, PI) at different doses of I-CRP after 24 h of treatment (Fig. 2). In healthy cells, PS is generally restricted to the inner leaflet of the cell membrane, and the exposure of phosphatidylserine on the outer leaflet is an effect that is commonly observed during apoptosis [15]. We determined PS externalization by flow cytometry of Annexin V-APC/Propidium Iodide-labelled cells that were treated with IMMUNEPOTENT CRP at different doses for 24 h. As shown in Fig. 2, I-CRP induced a slight population of AnnexinV-positive and PI-negative cells and most of them were double positive cells. Furthermore, as expected with MTT results, I-CRP induced cell-death in a concentration-dependent manner. It provoked a slight cell death induction at 0.75 U/mL, reaching 35% at 1 U/mL. At a 1.25 U/mL dose it induced cell death in 50% of the cells, increasing near to 80% at a 1.5 U/mL dose, and reaching complete cell death at 2 U/mL. Additionally, when we assessed trypan blue exclusion using 1.25 U/mL after a treatment of 16 h, 24 h, 48 h, and 72 h, we saw that it occurs at a time-dependent manner, reaching complete cell death after 48 h of treatment (Fig. 3a).

Next, we assessed cell recovery after different times of 1.25 U/mL treatment with I-CRP and then treatment-withdrawal. The cells were treated for 16 h, 24 h, 48 h, or 72 h, then washed and reseeded for 24 h. We observed that the cells were already compromised, as they did not recover from the MTT reduction, maintaining the same relative viability as if the treatment had not been removed (Fig. 3b, 1). Moreover, morphological assessment showed a reduction of cell confluence and alterations in cell morphology that were visible after 16 h of treatment and these alterations increased through time, reaching a complete cell loss after 72 h of I-CRP- treatment (Fig. 3C).

### IMMUNEPOTENT CRP induces cell cycle arrest in HeLa cells

**Fig. 4:**
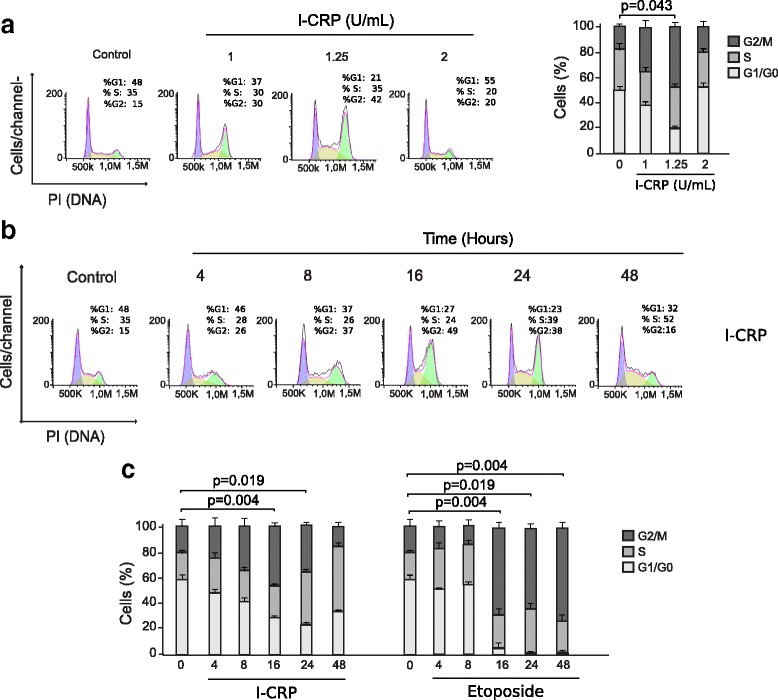
Cell cycle changes after I-CRP treatment of HeLa cells. **a** Schematic representation of changes in cellular DNA content in cells treated with different concentrations of I-CRP (1, 1.25, 2 U/mL) for 24 h, stained with propidium iodide and analyzed by flow cytometry to determine the cell-cycle distribution (left). The results were analyzed using Flowjo software and graphed (right). **b** Cell-cycle distribution of cells treated with I-CRP (1.25 U/mL) for different times (4, 8 16, 24, 48 h). **c** Cells were treated with I-CRP (1.25 U/mL) or etoposide (100 μM) for different times (4, 8 16, 24, 48 h) and analyzed as in (A) and graphed

Interestingly, although MTT showed that the CC50 after 24-h treatment was 1 U/mL, we confirmed that it is necessary to use 1.25 U/mL of I-CRP to induce cell death. Because the decrease in MTT activity can be due to cell death and/or to the decrease of cell division, we further assessed cell cycle after I-CRP treatment, to determine if it induces cell cycle arrest. A seen in Fig. 4, I-CRP effectively induces cell cycle arrest in G2/M phase at 1 U/mL, which also increases at a concentration of 1.25 U/mL. We could not detect cell cycle arrest, at a concentration of 2 U/mL probably because cell death was directly induced at this concentration, as shown in Fig. 2a.

To verify that cell cycle arrest was time-dependent, we used 1.25 U/mL treatment and assessed cell cycle arrest after 4, 8, 16, 24, and 48 h of treatment. At 4 h of treatment we noticed a slight accumulation of cells in G2/M phases that increased with time and reached the maximum accumulation after 24 h of treatment, then decreased after 48 h (Fig. 4b, c). We used Etoposide as a positive control, as it is a topoisomerase II inhibitor that induces p53-dependent cell cycle arrest in G2/M phase [16], and we saw a more potent cycle arrest in G2/M phase at 16, 24, and 48 h of treatment (Fig. 4c). Although both treatments induce cell cycle arrest in G2/M phase, I-CRP induces this arrest earlier and it probably does it in a different manner.

### IMMUNEPOTENT CRP induces DNA alterations

**Fig. 5:**
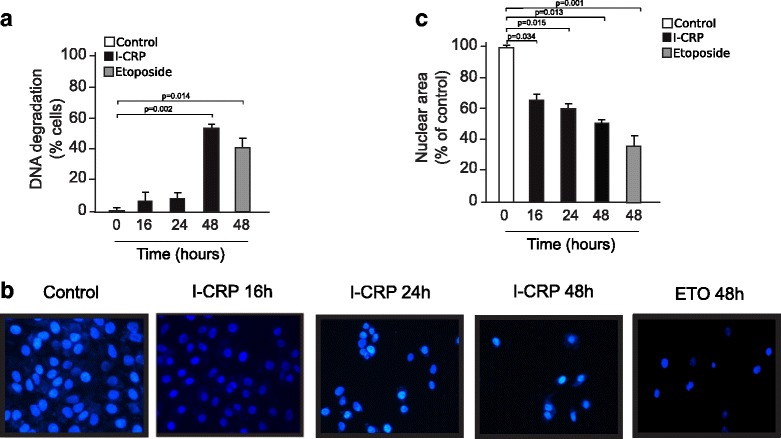
Nuclear alterations induced by I-CRP in HeLa cells. **a** Degradation of DNA in Cells treated with I-CRP (1.25 U/mL) for 16 h, 24 h, 48 h and 72 h or Etoposide for 48 h. **b** Nuclear size measured by Image-J software of cells treated with I-CRP for different times (16, 24, 48 h), and stained with Hoechst 33,342. The percentages refer to nuclear size represent as % control (non-treated nuclear size = 100%). **c** Nuclear morphology of cells treated with I-CRP for 16 h, 24 h and 48 h or etoposide (100 μM for 48 h), stained with Hoechst 33,342 and visualized by fluorescence microscopy (OLYMPUS IX70) (40X)

One of the major features of regulated cell death is the degradation of DNA and nuclear condensation. Different types of endonucleases activated during many types of RCD cleave sections of internucleosomal DNA and cause extensive DNA fragmentation [17]. The fragmented, low molecular weight DNA can be extracted from the cells during the process of cell staining, and cells with fractional, sub-G1, DNA content can be quantified. To assess DNA degradation, we quantified sub-G1 population of cells treated at different times with I-CRP. As shown in Fig. 5a, Sub-G1 was detected after 48 h of I-CRP treatment, indicating the late stages of cell death, similar to the effect observed by Etoposide treatment. To further characterize these nuclear alterations, we stained HeLa cells with Hoechst and nuclear morphology was assessed using a fluorescence microscope. As shown in Fig. 5b, I-CRP–treated cells show an altered nucleus after 16 h of treatment, and a type-2 necklace condensation [18], after 48 h, while Etoposide treatment shows a higher DNA condensation (Fig. c). Altogether, these results demonstrate that I-CRP induces DNA degradation and a partial chromatin condensation, indicating that nuclear alterations are a late step in I-CRP induced cell death.

### IMMUNEPOTENT CRP induces caspase-independent cell death

**Fig. 6:**
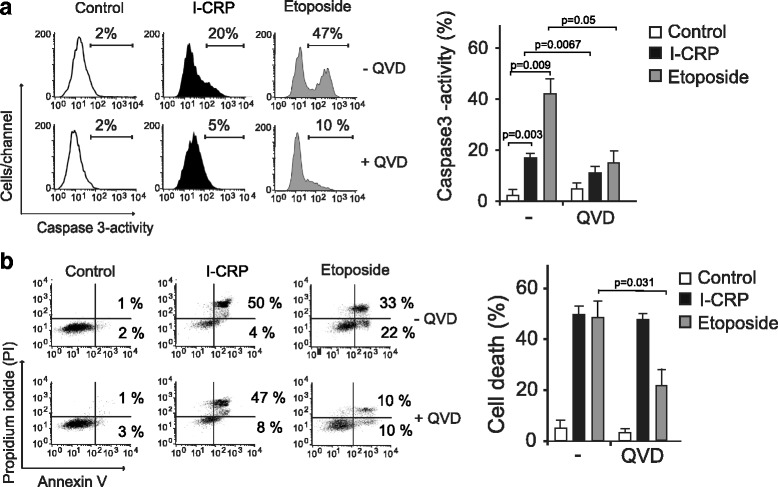
Caspase-3 activity and effects of pan-caspase inhibition on HeLa cells treated with I-CRP. **a** Caspase-3 activation was measured by flow cytometry in cells that were left alone or pretreated with QVD.oph and then treated with I-CRP (1.25 U/ml) or etoposide (100 μM) for 24 h, data was then analyzed and graphed. **b** Cell viability was determined by flow cytometry in cells that were left alone or pretreated with QVD.oph and then treated with I-CRP (1.25 U/ml) or etoposide (100 μM) for 24 h. The percentages refer to Annexin-V-positive or Annexin-V-positive/PI-positive staining

To verify the main molecular regulators of this type of cell death, we assessed caspase activity. As shown in Fig. 6, I-CRP induces slight caspase activation, as determined by the detection of caspase-3 activation (A). To determine if this type of cell death was dependent on caspase activity we used the pan-caspase inhibitor QVD.oph [19] and we found that, contrary to etoposide treatment, I-CRP-mediated cell death was independent of caspase-activation (Fig. 6b), in fact, the use of this pan-caspase inhibitor blocked caspase activation and etoposide-induced apoptosis but it did not inhibit the cell death induced by I-CRP (Fig. 6b). This result shows that although a small percentage of caspase-3 is activated by I-CRP, caspase-activity is not necessary for I-CRP-induced RCD.

### IMMUNEPOTENT CRP induces loss of mitochondrial membrane potential and ROS-dependent cell death

**Fig. 7:**
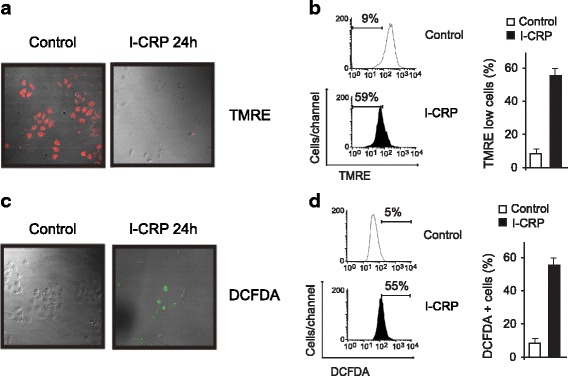
Mitochondrial membrane potential and ROS production of HeLa cells treated with I-CRP. **a** Mitochondrial membrane potential was measured by fluorescence microscopy (25X) using TMRE staining in non-treated cells (control) or treated with I-CRP (1.25 U/mL). **b** Mitochondrial membrane potential was measured by flow cytometry through TMRE staining in cells left alone or treated with I-CRP (1.25 U/mL) for 24 h. **c** ROS levels were measured by fluorescence microscopy (25X) using DCFDA staining in non-treated cells (control) or treated with I-CRP (1.25 U/mL). **d** ROS levels were measured by flow cytometry through DCFDA staining in cells left alone or treated with I-CRP (1.25 U/mL) for 24 h

The role of mitochondria in cell death is widely accepted, as they play a central role in cellular energetics and cell death signaling [20]. Moreover, mitochondrial dysfunction leads to reactive-oxygen species (ROS) generation [21–23], which has been associated with caspase-independent types of cell death [24]. As we found that caspases are dispensable for this type of cell death, we assessed whether the I-CRP was able to induce loss of mitochondrial membrane potential and ROS production, through tetramethylrhodamine ethyl ester (TMRE) and 2′,7′-dichlorofluorescin diacetate (DCFDA) staining, followed by fluorescence microscopy and flow cytometric analysis. As shown in Fig. 7, I-CRP induces loss of mitochondrial membrane potential and ROS production, as shown by fluorescence microscopy (7A,C) and flow cytometry (7B,D), in HeLa cells.

**Fig. 8:**
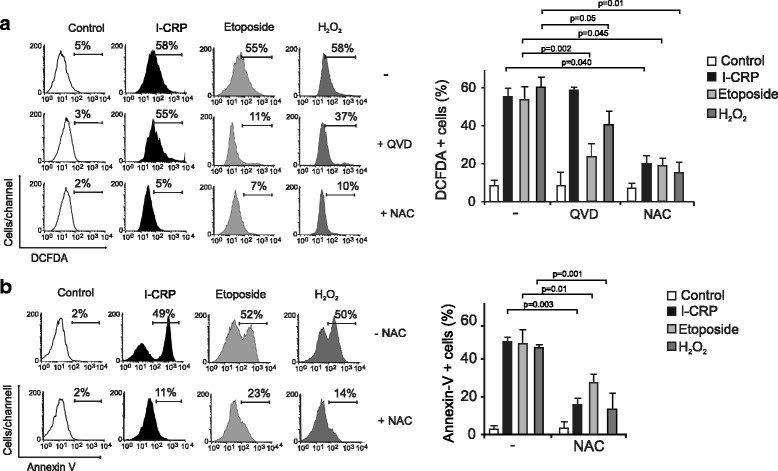
ROS production and the effect of their inhibition upon I-CRP-treatment of HeLa cells. **a** ROS levels were measured by flow cytometry through DCFDA staining in cells left alone or pretreated with NAC or QVD.oph and then treated with I-CRP (1.25 U/mL), etoposide (100 μM), or H_2_O_2_ (100 μM) for 24 h. **b** The effect on cell viability of cells left alone or pretreated with NAC and then treated with I-CRP (1.25 U/mL), etoposide (100 μM), or H_2_O_2_ (100 μM) for 24 h, was analyzed by flow cytometry through Annexin-V staining. The results were analyzed using FlowJo software and graphed

Moreover, I-CRP- treatment induces ROS production in a level similar to Etoposide and H_2_O_2_ at their CC50 (8A). We also found a correlation between ROS production and PS exposure, because approximately 50% of the cells display these two features (Fig. 8). Moreover, when we use QVD we can see that it is not able to inhibit ROS production in I-CRP treated cells, while it inhibits its production in Etoposide-treated cells and partially in H_2_O_2_-treated cells (Fig. 8a). Then, we used the antioxidant N-acetyl-L-cysteine (NAC), which increases intracellular GSH levels and possesses thiol-disulfide exchange activity [25, 26], to determine if it was able to inhibit ROS production. As shown in Fig. 8a, NAC was able to inhibit ROS production induced by I-CRP, Etoposide, and H_2_O_2_.

Finally, to determine if ROS were playing a role in I-CRP-induced cell death, we pretreated cells with NAC, before treating them with I-CRP. As shown in Fig. 8b, NAC was able to inhibit I-CRP induced cell death in HeLa and SiHa (Additional file 1: Fig. S1) cells, as observed by the reduction of Annexin V+ staining. The same result was observed when assessing H_2_O_2_-treated cells previously incubated with NAC and partially in Etoposide-treated cells (Fig. 8b).

NAC also inhibited caspase-3 activation in I-CRP-treated cells (Additional file 2: Fig. S2), which indicated that caspase-3 activation is a secondary effect of ROS production after I-CRP treatment.

Overall these results show that I-CRP induces a regulated type of cell death that is independent of caspase-3 activation, and induces cell cycle arrest, DNA fragmentation, mitochondrial damage, and ROS-dependent cell death.

## Discussion

Conventional therapies used against cancer, including cervical cancer [27, 28], exhibit many side effects due to their lack of specificity to cancer cells, while recently developed approaches have shown a limited success [29–31]. Because of this, the search for new therapies that improve existing treatments has become an important subject of study.

Dialyzable leukocyte extracts containing transfer factors have been used as adjuvants for chemotherapy for osteosarcoma [32], prostate cancer [33], lung cancer [34, 35], and breast cancer [9]. In these cases the extract has proven to be beneficial for cancer patients by improving their immune system by increasing cell-mediated cytotoxicity [32] or leukocyte cell number [9, 35], resulting in higher survival rates in patients treated [12]. Furthermore, it has also been demonstrated that dialyzable leukocyte extracts alone can be cytotoxic to cancer cells [5, 36–39], which has been specially studied using the bovine dialyzable leukocyte extract, IMMUNEPOTENT-CRP [6, 8, 10]. However, the mechanism underlying cell viability reduction by dialyzable leukocyte extracts, including I-CRP is misunderstood. Although several studies were done to understand this mechanism, no principal effector has been found.

In the present study, we showed that I-CRP reduced cell viability of HeLa cells by inducing cell cycle arrest and cell death. I-CRP induced changes in cell morphology, and decreased cell viability in a time and concentration-dependent manner, and we realized that this decrease was due to both cell death and cell cycle arrest. Cell cycle was arrested in phase G2/M and reached its maximum at 1.25 U/mL, and after 16 and 24-h of treatment. We also observed a low caspase-3 activity generated by I-CRP, however this activity is not necessary for the execution of cell death, because cell death occurred even after the activation of caspase-3 was inhibited by the pan caspase inhibitor QVD.Oph. DNA alterations, including chromatin condensation and DNA degradation, were observed as a late step of cell death induction, as they are observed after 48 h of treatment, when most of the cells have undergone membrane permeability loss. We further showed that I-CRP induced ROS production in a caspase-independent manner, and that this production is indispensable for cell death induction.

The past decade has witnessed a steady accumulation of findings leading to the description of many cancer cell death pathways, opening the possibilities to eradicate apoptosis-resistant cells. These findings also suggest that multiple cell death modalities can engage common sub-cellular sites and organelles, and even share initiator and effector molecules [40, 41].

Caspase-independent cell death has been observed in a variety of cancer treatments, such as ionizing radiation [42], CD47 agonist peptides [13], berberine [43], differentiation inducing factor 3 [44], geranylated 4-phenylcoumarins [45], among others. Interestingly, this type of cell death could be used to prevent apoptosis resistance in tumor cells. In some cases, such as I-CRP, there is an activity of caspases but this activity is not necessary to carry out the cell death, this is because the caspases can be involved in non-lethal cell processes such as differentiation, normal cell signalling and maturation [46], in addition to their immune functions [47]. Like I-CRP other agents also induce activation of caspases but kill cancer cells through a caspase-independent mechanism, such it is the case of bisanthracycline WP 631 [48], selenosemicarbazone metal complexes [49], phenoxazine derivatives [50], among others. Many of these agents induce caspase-independent cell death mechanisms that have been well documented and whose characterization has helped to determine shared features of these types of cell death modalities, such is the case of matrine [51] and methylnitronitrosoguanidine (MNNG) [52, 53], that induce mitochondrial damage, and subsequently AIF-dependent cell death.

Cell cycle arrest in G1 phase [54–57], S phase [58], or G2/M phase [59, 60] has commonly been observed as an early step in several types of cell death [61–63]. Here we show that cell cycle was arrested in G2/M phase, and this was observed from 8 h to 48 h of treatment at a 1.25 U/mL concentration. There are several agents, such genistein [64], austrobailignan-1 [65], and curcumin analog WZ35 [66], that can also cause an arrest in the G2/M phase of the cell cycle followed by cell death induction. Although a recent publication of our research group showed that I-CRP does not affect cell cycle in bone marrow cells of mice treated with I-CRP [11], here we show that in cervical cancer cells it induces cell cycle arrest in G2/M phase, uncovering a different effect in cancer cells.

DNA degradation has become a crucial target for cell death induction, however, we should consider that blockage of DNA-degrading enzymes does not prevent cell death during apoptosis, and enucleated cytoplasts can be induced to undergo apoptosis [67], indicating that the nucleus is not always a prime target of apoptosis and cytoplasmic process can play a major role in the programmed cell death initiation. Here, we observed DNA alterations induced by I-CRP, including partial chromatin condensation and DNA degradation as a late step of cell death induction (after 48 h of treatment) when most of the cells are dead, indicating that, DNA degradation does not play a role in this type of cell death, but it is a consequence of this process.

ROS are produced as a result of cellular metabolism at low-to-moderate concentrations and participate in physiological cell processes. However when produced at high concentrations, they produce adverse modifications to cell components, such as lipids, proteins, and DNA, affecting cellular organelles and functions and leading to cell death [68–72]. Caspase-independent cell death has been commonly associated with production of ROS [73, 74]. Some of these types of cell death can be associated with ROS, but independent of their production [75], or caspase-independent and ROS-dependent, such is the case of as Mn porphyrin in combination with ascorbate [76] and Obinutuzumab [77]. Yet, the cell death modalities that are ROS-dependent can be very vast and include autophagy, necrosis, pyroptosis, and mitoptosis [23, 70]. As the cell death induced by I-CRP was ROS-dependent and the alterations in cell morphology seemed to include cytoplasmic vacuolization, autophagy could be an interesting clue to follow, to better characterize the cell death mechanism induced by I-CRP.

Recently, our research group has published that I-CRP possess antioxidant capacity in lipopolysaccharide-stimulated human blood cells [78], murine peritoneal macrophages [79], and in bone marrow cells of 5-fluoracil-treated mice [11]. Here we show for the first time that I-CRP provokes ROS production in cancer cells, which are required for cell death induction. These results hint at a complex mechanism by which I-CRP induces a selective cell death depending on the type of cells. However, further studies are necessary to understand the differences of the cell mechanisms activated in different cell types.

The results shown here suggest that I-CRP can display multiple effects on cancer cells, yet ROS production was found to be indispensable for cell death. Many studies have involved ROS production in a variety of cell death modalities [23, 24, 69, 80, 81], and they can act as either initiators or executioners of cell death [24, 40, 68]. Additional studies must be done to understand how ROS are produced after I-CRP treatment in HeLa cells and their role as initiators or executioner of cell death induced by I-CRP.

## Conclusions

In conclusion, I-CRP treatment in HeLa cells diminishes cell viability through cell death and cell cycle arrest, which is accompanied by DNA alterations in the late steps of this type of RCD. Interestingly, caspase-3 was modestly activated after I-CRP treatment, yet RCD was independent of caspase-activity. Furthermore, I-CRP treatment induces loss of mitochondrial membrane potential and ROS production. The use of the antioxidant NAC prevents ROS and cell death induced by I-CRP, indicating that I-CRP induces ROS-dependent cell death. Overall, this work sheds light into the regulated pathway by which I-CRP reduces cell viability in HeLa cells, uncovering a cell death modality that dispenses of caspase activation. This work opens the way to further analyze the characterization of this atypical type of cell death that might be activated in parallel with apoptosis-inducing chemotherapies.

## Additional files

Additional file 1: Figure S1. (A) ROS levels were measured by flow cytometry through DCFDA staining in SiHa cells left alone or pretreated with NAC or QVD.oph and then treated with I-CRP (1.25 U/mL) for 24 h. (B) The effect on cell death of cells left alone or pretreated with NAC or QVD.oph and then treated with I-CRP (1.25 U/mL) for 24 h, was analyzed by flow cytometry through Annexin-V staining. The results were analyzed and graphed. (PDF 20 kb)

Additional file 2: Figure S2. Left, caspase-3 activity of HeLa cells left untreated or pretreated with Nac, and then treated with I-CRP. Right, the results obtained were analyzed and graphed as the percentage of HeLa cells positive for caspase-3 activity. (PDF 37 kb)

## Abbreviations

Ann/PI: Annexin-V-Allocp/ Propidium iodide.
I-CRP: Immunepotent-CRP
RCD: Regulated cell death
