# Supplementary figures and images for: IMMUNEPOTENT CRP induces cell cycle arrest and caspase-independent regulated cell death in HeLa cells through reactive oxygen species production
Source: BMC Cancer. 2018 Jan 3;18:13. doi: 10.1186/s12885-017-3954-5 (PMC5753472; doi:10.1186/s12885-017-3954-5)

**A**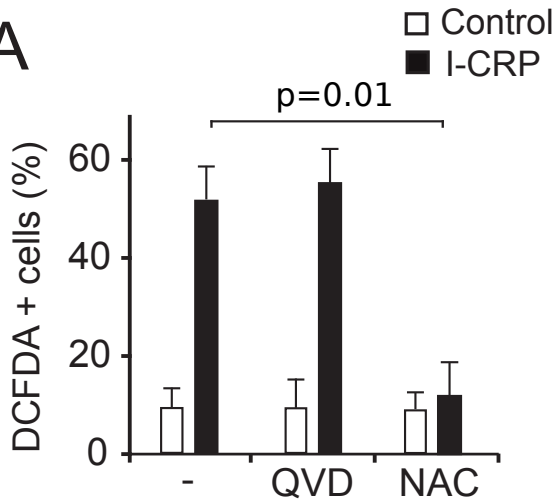**B**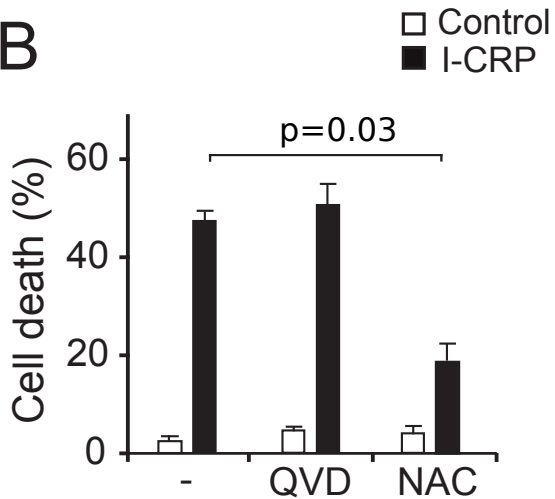

Supplement: Supplementary file 1 — (A) ROS levels were measured by flow cytometry through DCFDA staining in SiHa cells left alone or pretreated with NAC or QVD.oph and then treated with I-CRP (1.25 U/mL) for 24 h. (B) The effect on cell death of cells left alone or pretreated with NAC or QVD.oph and then treated with I-CRP (1.25 U/mL) for 24 h, was analyzed by flow cytometry through Annexin-V staining. The results were analyzed and graphed. (PDF 20 kb) [file 12885_2017_3954_MOESM1_ESM.pdf]

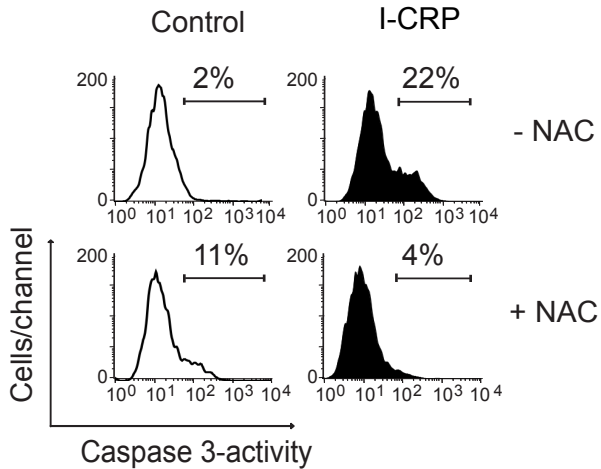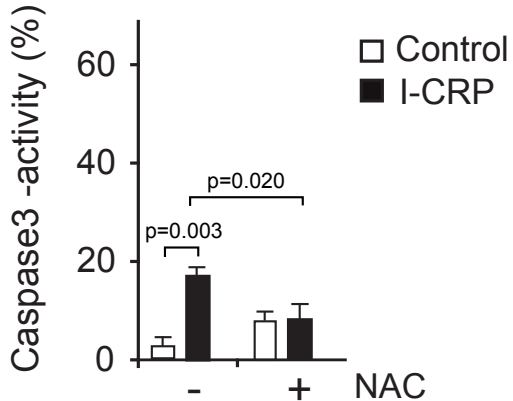

Supplement: Supplementary file 2 — Left, caspase-3 activity of HeLa cells left untreated or pretreated with Nac, and then treated with I-CRP. Right, the results obtained were analyzed and graphed as the percentage of HeLa cells positive for caspase-3 activity. (PDF 37 kb) [file 12885_2017_3954_MOESM2_ESM.pdf]
